# 311 disaster and mental health countermeasures

**DOI:** 10.3402/ejpt.v5.26515

**Published:** 2014-12-09

**Authors:** Yoshiharu Kim

**Affiliations:** National Center of Neurology and Psychiatry, Tokyo, Japan

**Keywords:** earthquake, tsunami, mental health policy, emergency response, psychosocial support, disaster

## Abstract

On 11 March 2011, a devastating earthquake struck off the coast of Japan, causing blustering tsunami that swept over the northeast coast of the country. Many struggled to evacuate from their homes, schools, and workplaces as 8- to 9-m-tall tsunami rapidly reached the coast within half an hour after the earthquake (Emergency Disaster Response Headquarters). The officials reported a record-breaking magnitude of 9.0 Mw, which made this earthquake the most devastating earthquake in the Japan's history. It had not been long since the previous massive earthquake had hit Kobe in 1995, killing 6,434 people (Japan Meteorological Agency). The author presents the outline of the initial mental-health-care responses at various levels. It has focused on the comprehensive strategies and policies that were intended to cover all the affected areas but has not described the individual countermeasures and reactions in each prefecture and city. The psychological effects of the atomic plant accident in Fukushima has not been mentioned in detail, because the scope of the physiological effect of the accident has not been settled yet and the society is not necessarily ready to deal with the accident as a psychological matter rather than a sociopolitical one. A number of psychiatric professionals are deeply concerned with the psychological and prolonged impact of the accident, including those who are in the Fukushima prefecture and conducting heroic efforts to care for the residents.

